# A population-based assessment of metastatic hepatoblastoma in Texas reveals ethnic disparities

**DOI:** 10.3389/fpubh.2023.1049727

**Published:** 2023-02-21

**Authors:** Andres F. Espinoza, Michael E. Scheurer, Tiffany M. Chambers, Sanjeev A. Vasudevan, Philip J. Lupo

**Affiliations:** ^1^Divisions of Pediatric Surgery and Surgical Research, Michael E. DeBakey Department of Surgery, Pediatric Surgical Oncology Laboratory, Texas Children's Surgical Oncology Program and Liver Tumor Program, Dan L. Duncan Cancer Center, Baylor College of Medicine, Houston, TX, United States; ^2^Department of Pediatrics, Hematology-Oncology Section, Texas Children's Hospital, Dan L. Duncan Cancer Center, Baylor College of Medicine, Houston, TX, United States

**Keywords:** hepatoblastoma, metastatic, race, Latino, disparities

## Abstract

**Background:**

Hepatoblastoma (HB) is the most common primary liver cancer in children with emerging evidence that incidence is increasing globally. While overall survival for low risk hepatoblastoma is >90%, children with metastatic disease have worse survival. As identifying factors associated with high-risk disease is critical for improving outcomes for these children, a need for a further understanding of the epidemiology of hepatoblastoma is warranted. Therefore, we conducted a population-based epidemiologic study of hepatoblastoma in Texas, a large state characterized by ethnic and geographic diversity.

**Methods:**

Information on children diagnosed with hepatoblastoma at 0–19 years of age for the period of 1995–2018 was obtained from the Texas Cancer Registry (TCR). Demographic and clinical variables including sex, race/ethnicity, age at diagnosis, urban-rural status, and residence along the Texas-Mexico border were evaluated. Multivariable Poisson regression was used to calculate adjusted incidence rate ratios (aIRRs) and 95% confidence intervals (CIs) for each variable of interest. Joinpoint regression analysis was used to determine the trend in incidence of hepatoblastoma, overall and by ethnicity.

**Results:**

Overall, 309 children diagnosed with hepatoblastoma in Texas for the period of 1995–2018. Joinpoint regression analysis showed no joinpoints in the overall or the ethnic-specific analyses. Over this period, the incidence increased at 4.59% annually; with the annual percent change higher among Latinos (5.12%) compared to non-Latinos (3.15%). Among these children, 57 (18%) had metastatic disease at diagnosis. Factors associated with hepatoblastoma included male sex (aIRR = 1.5, 95% CI: 1.2–1.8, *p* = 0.002); infancy (aIRR = 7.6, 95% CI: 6.0–9.7, *p* < 0.001); and Latino ethnicity (aIRR = 1.3, 95% CI: 1.0–1.7, *p* = 0.04). Additionally, children living in rural areas were less likely to develop hepatoblastoma (aIRR = 0.6, 95% CI: 0.4–1.0, *p* = 0.03). While residence on the Texas-Mexico border association with hepatoblastoma approached statistical significance (*p* = 0.06) in unadjusted models, this finding did not remain significant after adjusting for Latino ethnicity. The two factors associated with being diagnosed with metastatic hepatoblastoma included Latino ethnicity (aIRR = 2.1, 95% CI: 1.1–3.8, *p* = 0.02) and male sex (aIRR = 2.4, 95% CI: 1.3–4.3, *p* = 0.003).

**Conclusions:**

In this large population-based study of hepatoblastoma, we found several factors associated with hepatoblastoma and metastatic disease. The reasons for a higher burden of hepatoblastoma among Latino children is unclear but could be due to differences in geographic genetic ancestry, environmental exposures, or other unmeasured factors. Additionally, it is notable that Latino children were also more likely to be diagnosed with metastatic hepatoblastoma compared to non-Latino white children. To our knowledge, this has not been previously reported and warrants further study to delineate the causes of this disparity and identify interventions to improve outcomes.

## Introduction

Hepatoblastoma (HB), is the most common liver malignancy in children, most commonly presenting in individuals under the age of 3 years (1). To treat patients with hepatoblastoma appropriately, they are risk stratified using age, alpha fetoprotein (AFP) levels, the extent of liver segment and blood vessel involvement, and the presence of metastatic disease (2, 3). Due to medical and surgical advancements that have occurred over the last few years, overall survival of low risk hepatoblastoma has increased from around 30% to more than 90% (4–8). High risk patients, including those with metastasis, older age, or bilobar liver involvement, have much lower survival rates; < 50% (2, 6–8). A combination of cisplatin/doxorubicin-based chemotherapy as well as an aggressive surgical approach for the liver tumor and metastatic sites has improved survival; however, there are still a significant number of children who succumb to treatment refractory or relapse disease.

Despite being a rare disease, hepatoblastoma makes up 1% of all solid tumor in children (3, 4). In 2007, Linabery and Ross (3) reported that the annual incidence of hepatoblastoma has increased to 4.3% (85% CI, 0.2%−8.7%) from 1992 to 2004. In 2019, Hubbard et al. (4) further demonstrated that there has been an increase in incidence of hepatoblastoma on a global scale in children under the age of 5. This has been historically true as we have also noticed a significant number of cases in our tertiary referral center in Texas (2–4). It is not apparent whether these changes in incidence have affected the population of Texas similarly or if there are any specific patient populations that are at higher risk. We sought to evaluate demographic, socioeconomic factors, and recent changes in incidence of all hepatoblastoma and metastatic hepatoblastoma.

## Methods

### Texas Cancer Registry study population

We included cases of hepatoblastoma identified in the Texas Cancer Registry (TCR) diagnosed at 0–19 years of age for the period 1995–2018. The TCR is one of the largest and most diverse population-based cancer registries with Gold Certification from the North American Association of Central Cancer Registries. We used the International Classification of Disease for Oncology, Third Edition (ICD-O-3) and included cases with a histology code of 8970, as defined by the International Classification of Childhood Cancer, Third Edition (ICCC-3) site group VII for hepatoblastoma. A total of 309 hepatoblastoma cases who fulfilled these criteria were identified in the TCR.

Hepatoblastoma cases were further categorized as localized vs. metastatic using the SEER summary stage variable from the TCR. Variables of interest obtained from the TCR included patient age at diagnosis (infancy defined as age < 1 year), biological sex, and race/ethnicity. We also obtained information on: (1) county of residence at the time of diagnosis; (2) rural vs. urban residence; and (3) residence along the Texas-Mexico Border.

### Statistical analysis

Descriptive statistics were evaluated for hepatoblastoma overall, as well as metastatic hepatoblastoma. We calculated incidence ratios for the entire population over the study period overall and by the following characteristics: infancy, biological sex, race/ethnicity, urban-rural residence, and residence along the Texas-Mexico border. Incidence ratios were age standardized and were calculated per million persons-years using the at-risk population present. To assess trends in hepatoblastoma incidence over time, joinpoint regression was used to calculate annual percent change (APC) in incidence, overall and by ethnicity. To assess factors associated with hepatoblastoma overall and metastatic hepatoblastoma, both crude incidence rate ratios (IRRs) and adjusted IRRs (aIRRs), as well as corresponding 95% confidence intervals (CIs), were estimated using Poisson regression. Joinpoint regresson analyses were conducted using the National Cancer Institute's Joinpoint Regression Program version 4.9.1.0 (9). All other analyses were conducted using Stata Version 15 (College Station, TX).

## Results

Demographic characteristics for hepatoblastoma overall and specifically metastatic disease are presented in Table 1. A total of 309 patients were identified in the TCR, 57 of whom were diagnosed with metastatic hepatoblastoma. Males were more commonly diagnosed with the general diagnosis of hepatoblastoma (60.2%) and metastatic hepatoblastoma (72.2%). The majority of patients diagnosed with hepatoblastoma were Latino (58.0%) while metastatic patients were also more commonly Latino (65.0%). When considering age at diagnosis, infants (i.e., < 1 year of age) made up 37.0% of all hepatoblastoma cases and 14.0% of all metastatic cases. Most of the patients diagnosed with hepatoblastoma and metastatic hepatoblastoma cases lived in urban areas and were not diagnosed along the Texas-Mexico border.

**Table 1:** Characteristics of hepatoblastoma and metabolic hepatoblastoma cases from the Texas Cancer Registry, 1995–2018.

**Variable**	**All hepatoblastoma**	**Metastatic hepatoblastoma**
**Sex**		
Female	123 (40%)	16 (28%)
Male	186 (60%)	41 (72%)
**Race/ethnicity**		
NH-White	102 (34%)	16 (28%)
Latino	174 (58%)	36 (65%)
NH-Black	23 (8%)	4 (7%)
**Infant**		
No	196 (63%)	49 (86%)
Yes	113 (37%)	8 (14%)
**Rural/Urban**		
Urban	286 (92%)	55 (96%)
Rural	23 (8%)	2 (4%)
**Texas-Mexico border residence**		
No	261 (84%)	53 (93%)
Yes	48 (16%)	4 (7%)

**Figure 1:**
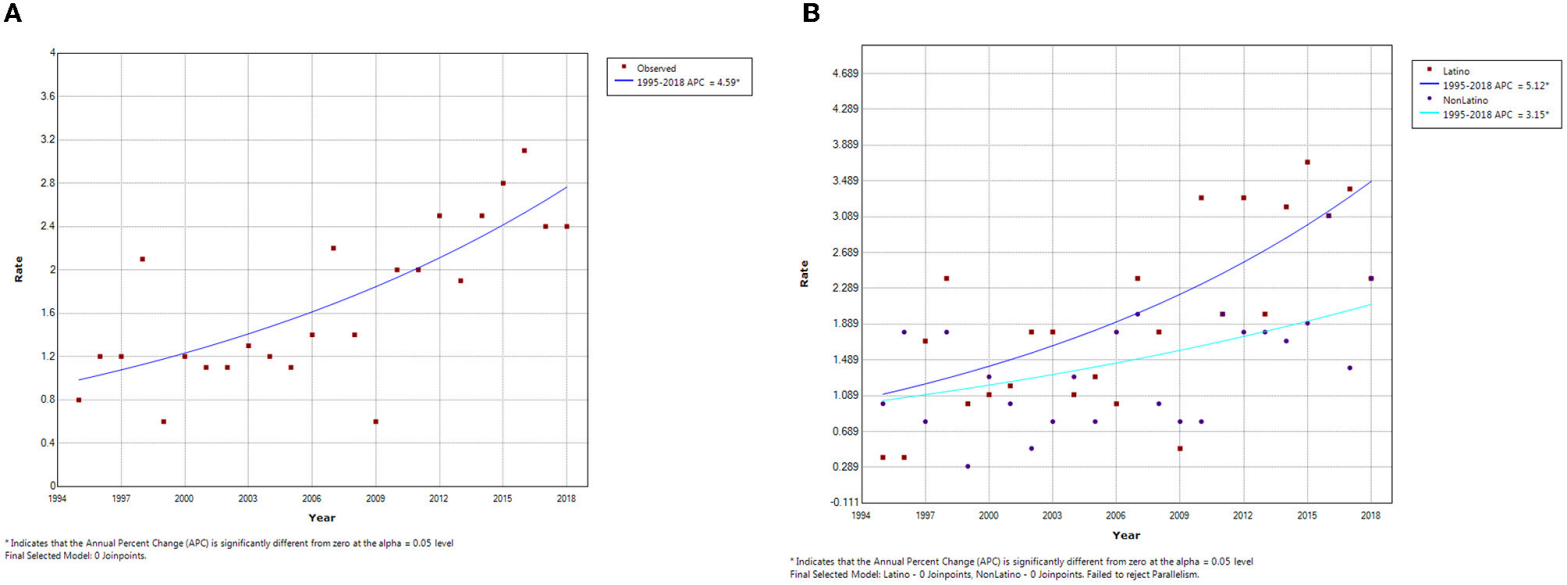
Joinpoint analysis of hepatoblastoma incidence trends in Texas, **(A)** Overall and **(B)** by Ethnicity, 1995–2018.

Joinpoint regression analysis did not identify any joinpoints in the overall or the ethnic-specific analyses. The overall incidence of hepatoblastoma increased from 0.8 per million in 1995 to 2.4 per million in 2018. This represents an APC of 4.59% (Figure 1A). This increase in incidence was more pronounced for Latinos (APC = 5.12%) compared to non-Latinos (APC = 3.15%; Figure 1B). Furthermore, based on a comparison of the incidence curves, the increase seems to be happening more sharply among Latinos, although a test for parallelism of the lines was not significant (*p* > 0.05).

The association between demographic factors and risk of hepatoblastoma are presented in Table 2. In crude analyses, hepatoblastoma was associated with infancy (IRR = 7.8, 95% CI: 6.1–9.8, *p* < 0.001), male sex (IRR = 1.4, 95% CI: 1.1–1.8, *p* = 0.002), and Latino ethnicity (IRR = 1.4, 95% CI: 1.1–1.8, *p* = 0.006). While not statistically significant, there was evidence that those living along the Texas-Mexico border were also more likely to develop hepatoblastoma (IRR = 1.3, 95% CI: 1.0–1.8, *p* = 0.06). Additionally, children in rural areas were less likely to develop hepatoblastoma compared to those in urban areas (IRR = 0.6, 95% CI: 0.4–1.0, *p* = 0.03). In adjusted analyses, the variables that remained significantly associated with hepatoblastoma included male sex (aIRR = 1.5, 95% CI: 1.2–1.8, *p* = 0.002), Latino ethnicity (aIRR of 1.3, 95% CI: 1.0–1.7, *p* = 0.04), infancy (aIRR = 7.6, 95% CI: 6.0–9.7, *p* < 0.001), and rural residence (aIRR = 0.6, 95% CI: 0.4–1.0, *p* = 0.03). Residence on the Texas-Mexico border was not found to be significantly predictive of incidence for hepatoblastoma on multivariate analysis (aIRR 1.1, 95% CI: 0.8–1.6, *p* = 0.448).

**Table 2:** Association between demographics characteristics and incidence of hepatoblastoma overall in Texas, 1995–2018.

**Variable**	**Crude**			**Adjusted**		
	**IRR**	**95% CI**	***p*** **-value**	**IRR**	**95% CI**	***p*** **-value**
**Sex**						
Female	1.0 (Ref)	1.0 (Ref)		1.0 (Ref)	1.0 (Ref)	
Male	1.4	1.1–1.8	**0.002**	1.5	1.2–1.8	**0.002**
**Race/Ethnicity**						
NH-White	1.0 (Ref)	1.0 (Ref)		1.0 (Ref)	1.0 (Ref)	
Latino	1.4	1.1–1.8	**0.006**	1.3	1.0–1.7	**0.040**
NH-Black	0.7	0.4–1.1	0.096	0.7	0.4–1.1	0.083
**Infant**						
No	1.0 (Ref)	1.0 (Ref)		1.0 (Ref)	1.0 (Ref)	
Yes	7.8	6.1–9.8	**< 0.001**	7.6	6.0–9.7	**< 0.001**
**Rural/urban**						
Urban	1.0 (Ref)	1.0 (Ref)		1.0 (Ref)	1.0 (Ref)	
Rural	0.6	0.4–1.0	**0.028**	0.6	0.4–1.0	**0.034**
**Texas-Mexico border residence**						
No	1.0 (Ref)	1.0 (Ref)		1.0 (Ref)	1.0 (Ref)	
Yes	1.3	1.0–1.8	0.060	1.1	0.8–1.6	0.448

The adjusted analysis controlled for all the variables mentioned in the tables. IRR, incidence rate ratio; CI, confidence interval; NH, non-Hispanic. Bold values indicates the statistically significant.

Associations between demographic features and metastatic hepatoblastoma are shown in Table 3. Overall, the only variables associated with metastatic hepatoblastoma in crude and adjusted models were male sex (aIRR = 2.4, 95% CI: 1.3–4.3, *p* = 0.003) and Latino ethnicity (aIRR = 2.1, 95% CI: 1.1–3.8, *p* = 0.02).

**Table 3:** Association between demographics characteristics and incidence of metastatic hepatoblastoma in Texas, 1995–2018.

**Variable**	**Crude**			**Adjusted**		
	**IRR**	**95% CI**	***p*** **-value**	**IRR**	**95% CI**	***p*** **-value**
**Sex**						
Female	1.0 (Ref)	1.0 (Ref)		1.0 (Ref)	1.0 (Ref)	
Male	2.4	1.3–4.3	**0.003**	2.4	1.3–4.3	**0.003**
**Race/ethnicity**						
NH-White	1.0 (Ref)	1.0 (Ref)		1.0 (Ref)	1.0 (Ref)	
Latino	1.9	1.0–3.4	**0.038**	2.1	1.1–3.8	**0.018**
NH-Black	0.8	0.2–2.3	0.615	0.7	0.2–2.1	0.546
**Infant**						
No	1.0 (Ref)	1.0 (Ref)		1.0 (Ref)	1.0 (Ref)	
Yes	2.0	0.9–4.3	0.096	1.9	0.8–4.2	0.110
**Rural/Urban**						
Urban	1.0 (Ref)	1.0 (Ref)		1.0 (Ref)	1.0 (Ref)	
Rural	0.3	0.1–1.1	0.074	0.3	0.1–1.2	0.091
**Texas-Mexico border residence**						
No	1.0 (Ref)	1.0 (Ref)		1.0 (Ref)	1.0 (Ref)	
Yes	0.5	0.2–1.5	0.236	0.4	0.1–1.1	0.075

The adjusted analysis controlled for all the variables mentioned in the tables. IRR, incidence rate ratio; CI, confidence interval; NH, non-Hispanic. Bold values indicates the statistically significant.

## Discussion

In our population-based assessment, we confirmed that the incidence of hepatoblastoma was increasing in Texas, and that this was particularly true for Latinos. We also found that infants, males, Latinos, and individuals in urban counties were more likely to develop hepatoblastoma. When considering the development of metastatic hepatoblastoma, factors associated with risk included male sex and Latino ethnicity.

Using data from SEER and other data sources, several previous assessments have concluded the incidence of hepatoblastoma is higher in males compared to females (9–21). Our findings support these observations and provide the novel finding that male sex is independently associated with metastatic hepatoblastoma. To our knowledge, one other study did observe a higher proportion of male patients in their cohort of metastatic patients, however, they did not evaluate a statistical difference (13). The role of biological sex in pediatric cancer (i.e., males are often more likely to develop pediatric cancer than females) has been well-described but deserves further attention. Many theories into the role of epigenetic alterations, autophagy, and other sex-linked oncogenic processes have been described in hepatoblastoma, especially metastatic disease (17–24). One prominent theory as to why males are at higher risk of hepatoblastoma is the onco-protective role of estrogen as it has been shown to decrease the level of bile acid, shown to be predictive in colon cancer but may play a role in liver cancer as well (17).

Another notable finding in our assessment was the higher incidence of hepatoblastoma among children who were Latino. In fact, we saw that the incidence among Latino children increased more rapidly than among non-Latino children during our assessment period. Latino ethnicity has been strongly associated with a higher incidence and morbidity in adult liver cancer (25–27). It is well recognized that Latino patients have more risk factors for liver disease and present with more aggressive disease than other ethnicities, however, overall survival does not appear to differ (25, 26). Despite these advances in understanding liver cancer in adults, there is limited data on the role that ethnicity has on incidence of hepatoblastoma. Some reports show that Latino and non-Latino White patients tend to have a higher frequency of the *Wnt/*β*-catenin* pathway mutation, the most common somatic mutation found in hepatoblastoma, which could point to differences in incidence (25, 27). Notably, our data suggest that Latinos not only have a higher burden of hepatoblastoma overall, but also of metastatic hepatoblastoma, even when adjusting for relevant covariates. Appropriate recognition of ethnicity as a risk factor is especially important for Latino patients given the predicted increase in percent of the overall U.S. population (26, 27). Given that our cohort shows the majority of metastatic hepatoblastoma cases are Latino, it is possible that the proportion of children with metastatic disease in the U.S. will increase as the proportion of individuals who identify as Latino continue to increase in the U.S.

According to the U.S Census Bureau, 11.9% of the U.S. population resided in mainly rural areas while 1.9% were completely rural (28–30). While population studies comparing these areas of residence in adult liver cancer have been described, pediatric liver cancer studies are lacking. In our assessment, we found that children living in rural areas were less likely to develop hepatoblastoma compared to those living in urban areas, but this was not the case for metastatic hepatoblastoma. In adult liver cancer, despite higher incidence in urban areas there appears to be a health disparity with patients in rural areas having worse outcomes (28–31). While it is important to acknowledge that there is a difference in incidence in pediatric cancer, further characterization of outcomes that these patients experience is warranted.

We next tried to identify if the incidence of hepatoblastoma was higher in counties along the Texas-Mexico border. While we did observe differences, they did not remain significant in multivariable models. Notably, there are reported differences between residents along the Texas-Mexico border compared to other regions in terms of cancer diagnosis and care (32, 33). Future assessments should evaluate outcomes in children diagnosed with hepatoblastoma in this region.

To our knowledge, this is the first study to demonstrate that male sex and Latino ethnicity are independent risk factors for metastatic hepatoblastoma. While many advances have been made with surgical and medical management, children with metastatic hepatoblastoma continue to have high morbidity and mortality (1–5). To treat patients with metastatic hepatoblastoma, further understanding of the incidence and demographic risk factors are warranted. Limited studies are available that look at the overall incidence of metastatic hepatoblastoma, especially in a diverse population as Texas, and with this study we provide another piece in understanding this high-risk disease.

## Data availability statement

The datasets presented in this study can be found in online repositories. The names of the repository/repositories and accession number(s) can be found in the article/supplementary material.

## Author contributions

All authors listed have made a substantial, direct, and intellectual contribution to the work and approved it for publication.
